# Multi-Type Microplastic Migration Model Driven by River Hydrodynamic Conditions

**DOI:** 10.3390/toxics14070600

**Published:** 2026-07-09

**Authors:** Yuxuan Li, Ming Dou, Xiaolu Li, Yongyong Zhang, Xueliang Cai, Zhen Wang

**Affiliations:** 1 School of Ecology and Environment, Zhengzhou University, No. 100 Kexue Road, Zhengzhou 450001, China; lyx0331@gs.zzu.edu.cn (Y.L.); xiaolu@gs.zzu.edu.cn (X.L.); 2 School of Water Conservancy and Transportation, Zhengzhou University, No. 100 Kexue Road, Zhengzhou 450001, China; fnfd@gs.zzu.edu.cn; 3 Key Laboratory of Water Cycle and Related Land Surface Processes, Institute of Geographic Sciences and Natural Resources Research, Chinese Academy of Sciences, Beijing 100101, China; zhangyy003@igsnrr.ac.cn; 4 Agriculture, Food, Nature, and Rural Development Sector Office, Sectors Group, Asian Development Bank, Mandaluyong City 1550, Philippines; yushufu0331@163.com

**Keywords:** microplastics, migration model, river hydrodynamic conditions, Mulanxi River

## Abstract

To address the difficulty in predicting the migration trajectories of microplastics in aquatic environments, this study develops a hydrodynamically driven migration model applicable to multiple types of microplastics. Based on hydraulic experiments, hydrodynamic thresholds are established to characterize transitions among drifting, suspension, and sedimentation. The model integrates hydrodynamic forces, gravity, buoyancy, and interparticle interactions, enabling accurate simulation of migration pathways and ultimate destinations. Compared with conventional models, the key innovation lies in incorporating differences in size, shape, and material, allowing differentiated representation and prediction of diverse microplastics. The pollutant accumulation patterns obtained by simulating microplastic migration in the Mulanxi River basin using this model are consistent with actual observational results, further demonstrating the model’s reliability and applicability. Results from the Xianyou Section show that microplastics smaller than 0.5 mm account for 71.62%, dominated by fragmentary and fibrous types. There are significant differences in the migration behaviour of microplastics made from different materials; these differences are primarily attributable to variations in their density and physicochemical properties. Furthermore, transport rates at the downstream end are positively correlated with proximity to pollution sources and the abundance of lightweight microplastics. The total flux reaches 9.37 × 10^11^ particles, with an overall transport rate of 68.34%. This study enhances the mechanistic understanding and predictive capability of microplastic transport in freshwater systems, providing new theoretical and methodological support for pollution control.

## 1. Introduction

Plastics have become indispensable in modern life due to their low cost and excellent physicochemical properties. However, China alone produced 33.3% of global plastics by 2023 [1], and vast quantities of plastic waste enter aquatic environments, where they fragment into microplastics (MPs, <5 mm) [2] through physical, chemical, and biological processes [3,4,5]. Due to their small size and large specific surface area, MPs act as carriers for toxic pollutants and threaten aquatic biota through chronic exposure [6]; their size, type, and shape jointly determine toxic bioaccumulation potential [7]. Understanding MP transport in rivers is therefore a prerequisite for assessing their environmental fate and ecological risks in freshwater ecosystems.

Rivers serve as both the primary recipients and transport pathways of MPs to marine environments [8], with 70~80% of marine MPs originating from inland freshwater systems [9]. MP migration is governed by hydrological characteristics, hydraulic conditions, and particle properties, including density, shape, and size [10]. Hydrodynamic conditions drive transport processes—drifting, suspension, sedimentation, and resuspension—and are consequently recognized as the dominant factor controlling MP flux and spatial distribution [11,12]. This spatial distribution, in turn, determines the exposure levels and ecological risks for aquatic organisms. Indeed, recent studies have documented toxicological effects from MP exposure, including oxidative stress, gut microbiota dysbiosis, immune dysfunction, and metabolic disorders [13,14,15,16]. Critically, hydrodynamic conditions also directly modulate MP bioaccumulation and toxicity [17], challenging static exposure paradigms and highlighting the need to incorporate hydrodynamics into risk assessments. This evidence underscores a critical gap: how hydrodynamic conditions shape the transport, distribution, and exposure risks of diverse MPs in real rivers remains poorly understood, motivating the hydrodynamically driven migration model developed in this study.

Recent advances in numerical modeling have improved our capacity to simulate MP transport. Eulerian approaches have been employed to predict MP distribution in lakes and coastal seas [18,19], while Lagrangian particle tracking models have successfully reproduced individual particle trajectories in open-channel flows [20]. Coupled CFD-DPM simulations have further enabled quantification of particle settling and horizontal migration velocities under controlled conditions [21]. Despite these contributions, existing models suffer from several critical limitations: they generally oversimplify particle diversity by neglecting shape-specific behaviors, derive parameters primarily from idealized laboratory experiments that may not represent complex field conditions, and often lack rigorous validation against field observations [22,23]. These deficiencies point to an urgent need for a more comprehensive modeling framework that explicitly accounts for the diversity of MP size, shape, and material composition while remaining robustly validated in real-world settings.

To address this gap, this study integrates hydraulic experiments, field investigations, and mathematical modeling to analyze MP behavior under varying hydrological conditions, elucidate the drivers of motion state transitions through single-particle force analysis, and simulate the migration of distinct MP types. The core objectives are to clarify riverine MP migration mechanisms governed by the interplay of particle properties and hydrodynamic forces, and to quantify movement pathways, depositional sinks, and transport fluxes. Validated against field observations from the Mulanxi River, the model supports watershed-scale risk assessment and pollution mitigation while establishing a mechanistic framework for forecasting MP transport in freshwater systems—thereby linking dynamic migration patterns to their associated ecotoxicological outcomes.

## 2. Motion Principle, Model Construction, and Experimental Setup of MPs

### 2.1. Analysis of the Motion Principle of MPs in Water

**Figure 1:**
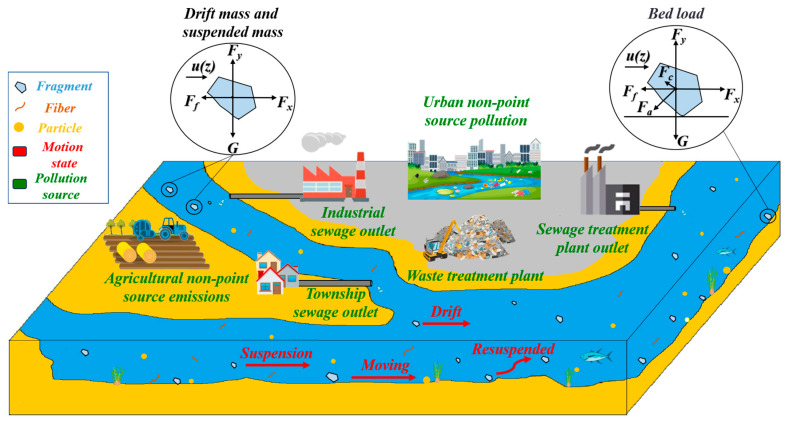
The main source of MPs and its force analysis and motion principle diagram.

The migration of MPs in natural water involves both horizontal and vertical movements. Horizontally, MPs may drift, suspend, or moving along the bed; vertically, processes include settlement and resuspension. Based on sediment transport principles [24], the existence state of MPs in water can be divided into drift mass, suspended mass, or bed load [25]. Horizontal motion is primarily driven by drag force (*F_x_*) and flow resistance (*F_f_*) [26], whose magnitude depends on MP properties and flow velocity, increasing with faster currents [27]. Vertically, MPs are affected by gravity (*G*) and the resultant force of lifting force and buoyancy (*F_y_*) [28] with settling and resuspension influenced by particle size, shape, and local flow conditions. Larger MPs tend to settle more readily, whereas smaller ones remain suspended. Bed load MPs are additionally subject to centrifugal force (*F_C_*) and shear stress (*F_a_*) [29], promoting rolling and sliding along the bottom. Overall, MP movement results from the interplay between their physicochemical properties (size, shape, density) and hydraulic conditions. The migration mechanism is summarized in Figure 1.

### 2.2. Construction of MP Migration Models in Different Motion States

**Figure 2:**
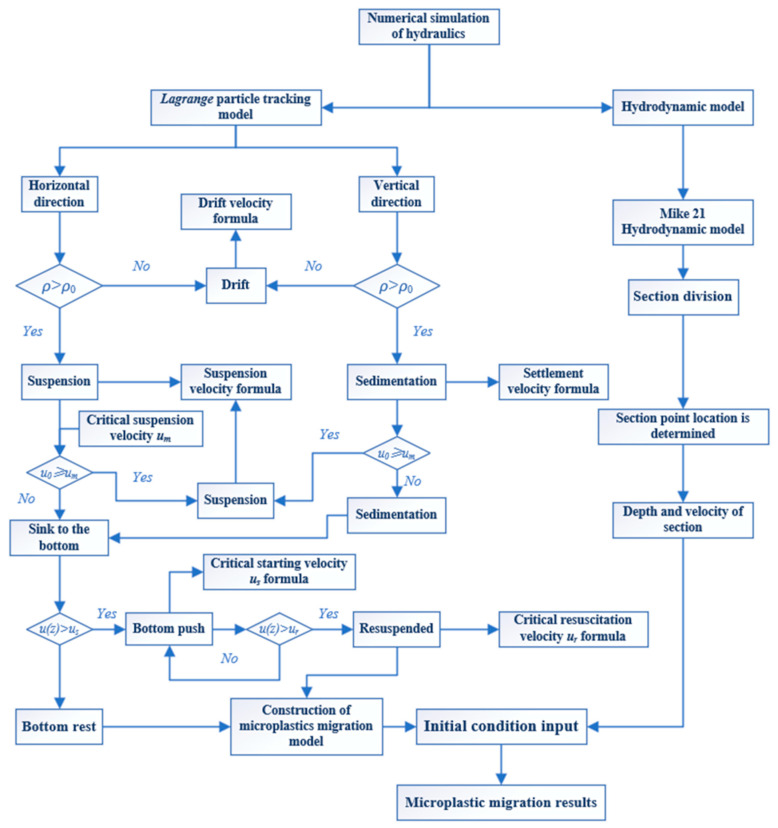
Schematic diagram of the model principle.

A river MP trajectory prediction model was developed by coupling the MIKE 21 hydrodynamic model with a physically based MP transport module, accounting for the diversity of MP motion states. The model addresses two key aspects: vertical movement differences under varying stress conditions, and the mathematical formulation of horizontal and vertical motions for different MP types. The overall model principle is illustrated in Figure 2.

The model incorporates key MP properties-density, particle size, and shape-to reflect individual differences and improve accuracy. It begins with a comprehensive force analysis for each MP, then determines its motion state (e.g., drift, suspension, or moving) based on internal properties (e.g., density) and external hydraulic conditions (e.g., flow velocity). Corresponding velocity expressions are derived for each state. Critical hydraulic thresholds (e.g., resuspension flow rate) obtained from flume experiments are used to judge state transitions. Once initial conditions are provided, the model can accurately predict the migration trajectories of different MPs.

#### 2.2.1. Hydrodynamic Drive Model Based on MIKE 21

In this paper, a two-dimensional MP migration model is developed and integrated based on the MIKE 21 platform. The model can effectively simulate the migration process of MPs by coupling hydrodynamics and a particle tracking algorithm, and it is consistent with the measured data to form an integrated simulation system. The water column was treated as vertically stratified to reflect real river conditions, and MP forces were analyzed in horizontal and vertical directions, accounting for factors such as density, size, shape, and flow velocity. The coupled model is derived from the 3D incompressible Navier–Stokes equations under Boussinesq and hydrostatic assumptions, yielding a 2D unsteady shallow-water equation set to simulate the hydrodynamically driven transport of MPs [30,31,32]:

(1) $$\frac{\partial h}{\partial t}+\frac{\partial h\bar{u}}{\partial x}+\frac{\partial h\bar{v}}{\partial y}=hS$$

(2) $$\frac{\partial h\bar{u}}{\partial t}+\frac{\partial h{\bar{u}}^{2}}{\partial x}+\frac{\partial h\bar{uv}}{\partial y}=f\bar{v}h-gh\frac{\partial \varepsilon }{\partial x}-\frac{h}{\rho_{0}}\frac{\partial \rho_{a}}{\partial x}-\frac{gh^{2}}{2\rho_{0}}\frac{\partial \rho }{\partial x}+\;\;\frac{\tau_{sx}}{\rho_{0}}-\frac{\tau_{bx}}{\rho_{0}}-\frac{1}{\rho_{0}}\left(\frac{\partial S_{xx}}{\partial x}+\frac{\partial S_{xy}}{\partial y}\right)+\frac{\partial }{\partial x}\left(hT_{xx}\right)+\frac{\partial }{\partial y}\left(hT_{xy}\right)+hu_{s}S$$

(3) $$\frac{\partial h\bar{v}}{\partial t}+\frac{\partial h\bar{uv}}{\partial x}+\frac{\partial h{\bar{v}}^{2}}{\partial y}=f\bar{u}h-gh\frac{\partial \varepsilon }{\partial y}-\frac{h}{\rho_{0}}\frac{\partial \rho_{a}}{\partial y}-\frac{gh^{2}}{2\rho_{0}}\frac{\partial \rho }{\partial y}+\;\frac{\tau_{sy}}{\rho_{0}}-\frac{\tau_{by}}{\rho_{0}}-\frac{1}{\rho_{0}}\left(\frac{\partial S_{yx}}{\partial x}+\frac{\partial S_{yy}}{\partial y}\right)+\frac{\partial }{\partial x}\left(hT_{xx}\right)+\frac{\partial }{\partial y}\left(hT_{xy}\right)+hv_{s}S$$

$$h\bar{u}=\int_{-o}^{\varepsilon }udz;$$

(4) $$h\bar{v}=\int_{-o}^{\varepsilon }vdz$$

$$T_{xx}=2A\frac{\partial \bar{u}}{\partial x};T_{xy}=A\left(\frac{\partial \bar{u}}{\partial y}+\frac{\partial \bar{v}}{\partial x}\right);$$

(5) $$T_{yy}=2A\frac{\partial \bar{v}}{\partial y}$$

where *t* is time; *x* and *y* are Cartesian coordinate system coordinates; *ε* is the water level; *o* is the static water depth; h=ε+0 is the total water depth; *u* and *v* are the velocity components in the *x* and *y* directions, respectively; *f* is the Coriolis force coefficient, f=2ωsinφ; *ω* is the angular velocity of the Earth’s rotation; φ is the local latitude; *g* is the acceleration of gravity; $\rho_{0}$ is the density of water; $S_{xx}$, $S_{xy}$ and $S_{yy}$ are radiation stress components, respectively. *S* is the source term; ($u_{s},\;v_{s}$) is the source term flow rate; $\bar{u},\bar{v}$ is the average velocity along the water depth; $T_{ij}$ is a horizontal viscous stress term, including viscous force, turbulent stress, and horizontal convection.

#### 2.2.2. MP Migration Model Based on Water Force Analysis

The Lagrangian particle tracking method is an effective method to study the migration of solid matter in a water environment. The basic principle is to simulate the advection transport process of particles by the drift mechanism and to simulate the diffusion process by a random motion term. The MP particles are regarded as the result of horizontal motion and vertical motion, and the following Lagrangian particle tracking model is established:

(6) $$\frac{dx}{dt}=U\left(x,t\right)+U^{\prime }\left(x,t\right)$$

where x is the particle coordinate; U is the migration velocity of particles in water flow; $U^{\prime }$ is the random velocity caused by the turbulence effect; t is time information.

At the same time, the measure formula of random motion is added to the model, and the following particle displacement equation is constructed [33]:

(7) $$\left\{\begin{array}{c} Horizontal\;direction:\;X^{t+1}=X^{t}+V∆t+R_{x}\sqrt{2\cdot D_{H}\cdot ∆t}\;{\;D}_{H}=0.6\left(h-z\right)u*\\ Vertical\;direction:\;Z^{t+1}=Z^{t}+v∆t+R_{z}\sqrt{2\cdot D_{v}\cdot ∆t}\;D_{v}=ku*z\left(1-\frac{z}{h}\right) \end{array}\right.$$

where $X^{t+1}$ and $X^{t}$ are the coordinates of the particles in the horizontal direction at t+1 and t, respectively; ∆t is the time step; *V* is the horizontal velocity vector of MP particles, m/s; R is a vector composed of independent random components with zero mean and unit variance, representing random motion ($R_{x}$ is a component along the *x* direction, and its range is set to [−1, 1]; $R_{z}$ is the component along *z* direction); *z* is the distance from the water surface to the location of the MP; $Z^{t+1}$ and $Z^{t}$ are the vertical coordinates of particles at time t+1 and t, respectively; *v* is the sedimentation velocity of MPs, m/s; $D_{v}$ is the vertical turbulent diffusion coefficient.

This study employed specific formulas to characterize three distinct MP motion states: drift (surface transport), suspension (particles suspended in the water column), and moving (rolling, saltation, and resuspension at the bed).

(1) Horizontal motion equation

The horizontal movement rate of drifting and suspended microplastic particles depends on the water flow rate at their location, and both follow a linear relationship. Therefore, in our calculations, we adopted the basic linear calculation to measure their horizontal movement rate. Formula (8) is established to calculate the drift or suspended velocity of MPs:

(8) $$V_{H}={ku}_{0}$$

where $V_{H}$ is the drift or suspended velocity, m/s; $u_{0}$ is the velocity of water flow, m/s; *k* is the drift or suspended coefficient.

The fitting of the formula for the horizontal velocity of the bed load is based on the calculation formula of the Shamov sediment transport velocity [24], and the formula for the transport velocity of the bed load MPs is obtained as follows:

(9) $$V_{p}=\left(u_{0}-\frac{u_{s}^{\prime }}{1.2}\right){\left(\frac{d_{s}}{h}\right)}^{0.25}$$

where $V_{p}$ is the velocity of displacement, m/s; h is the water depth, m; $u_{s}$ is the critical startup velocity, m/s; $d_{s}$ is the equivalent particle size, m; other symbolic meanings are the same as before.

According to the hydraulic experiment, the calculation formula for the critical startup velocity and the critical resuspension velocity of the MP particles is fitted:

(10) $$u_{s}=0.052{CSF}^{-0.295}\sqrt{\frac{\left(\rho_{s}-\rho_{0}\right)gd_{s}}{\rho_{0}}}{\left(\frac{d_{s}}{h}\right)}^{-0.663}$$

(11) $$u_{r}=9.848{{CSF}^{-0.063}\sqrt{\frac{\left(\rho_{s}-\rho_{0}\right)gd_{s}}{\rho_{0}}}\left(\frac{d_{s}}{h}\right)}^{0.019}$$

where $u_{s}$ is the critical startup velocity, m/s; $u_{r}$ is the critical resuspension velocity, m/s; $\rho_{s}$ is the density of MPs, kg/m^3^; $\rho_{0}$ is the density of water, kg/m^3^; CSF is the Corey Shape Coefficient of irregular particles, assuming that the long axis of the particle is *A*, the central axis is *B*, and the short axis is *C*, so $CSF=\frac{C}{\sqrt{AB}}$; other symbolic meanings are the same as before.

(2) Vertical motion equation

In the vertical direction, the vertical motion of MPs mainly considers the effects of gravity, buoyancy, lifting force, and vertical turbulence on MPs. Since the gravity of the drift mass is less than the combined force of buoyancy and lifting, it is assumed that the vertical movement is only affected by turbulence, that is the settlement velocity of the drift mass. $v_{f}=0$. The force of suspended MPs in the vertical direction changes greatly, which is related to the particle size, shape, and density of MPs themselves. According to the settlement experiment results, the settlement velocity formula of MPs is fitted [34,35]:

(12) $$v_{m}=A_{1}\left(1.0434{\left(\frac{\rho_{s}-\rho_{0}}{\rho_{0}}g\right)}^{0.495}\frac{{d_{s}}^{0.777}{CSF}^{0.710}}{{u\left(z\right)}^{0.124}}\right)$$

where $v_{m}$ is the sedimentation velocity of Suspended mass MPs, m/s.

The process of bed load from entering the water body to sinking into the bottom is the same as that of suspended mass. When the bed load sinks into the bottom of the water, the vertical position calculation depends on the relationship between the water’s bottom velocity, the critical startup velocity, and the critical resuspension velocity. Based on the above description, the formula for the bottom velocity of the bed load MPs is derived.

Different densities of MPs have different degrees of turbulence at different locations. According to the results of hydraulic experiments, the range of the drift mass $R_{z}$ is set to [−0.5, 0.5], the range of the suspended mass particle $R_{z}$ is set to [−1, 1], and the range of bed load particles $R_{z}$ is set to [−0.1, 0.1].

MP transport through the study area was assessed based on whether the horizontal travel distance within the set time exceeded the source-to-outlet distance. The model calculated this distance for all released MPs and compared it with the source-to-outlet distance to determine the transport rate—the proportion of MPs that transited the area. Transport rates were determined for all MP types using the following formula:

(13) $$\delta =\frac{n_{pass}}{n_{in}}*100\%$$

where δ is the transport rate of MPs; $n_{in}$ is the input of MPs, n; $n_{pass}$ is the amount of MPs finally passing through the study area, n.

### 2.3. Experiment Setting

The experimental part is mainly divided into the sample determination experiment and the hydraulic experiment:

The sample determination experiment was based on pre-arranged sampling points, and surface water and sediment were collected, respectively. The surface water samples were collected at the river edge at 3–5 m and at a water depth of 1 m. The samples were concentrated by a 180-mesh sieve, washed, and stored in marked shading bottles. Sediment samples were taken from a depth of 5 cm on the sediment surface, and MPs were separated by flotation in saturated NaCl solution, and the supernatant was collected. In order to reduce pollution, all containers and tools were cleaned with ultrapure water and sealed for storage, and two parallel samples were collected at each sampling point. After the samples were stored at 4 °C, MPs were extracted and purified by organic matter digestion, Nile red staining, and microporous filtration. The counting, shape analysis, and type identification of MPs were completed by RX50 microscope (Ningbo Sunny Instrument Co., Ltd., Ningbo, China), Image J 1.54p, and LabRAM HR Evolution Raman spectrometer (HORIBA France SAS, Palaiseau, France).

**Figure 3:**
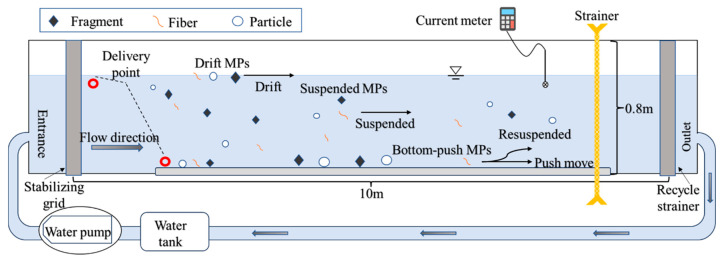
Flume experimental design diagram.

In the hydraulic experiment, five kinds of original plastics, PE, PS, PA, PET, and PVC, were selected and frozen in liquid nitrogen to grind into irregular MP particles with a particle size less than 5 mm. The whole experiment is carried out in a closed, self-circulating flume which is equipped with a water tank, a stabilizing grid, a recycle strainer, and an artificial riverbed. The experimental design of the flume is shown in Figure 3. Single MPs were placed from the water surface and the bottom, respectively, and the flow rate was gradually increased. The instantaneous flow rate and the migration rate under different conditions were recorded when the motion state changed critically. The data statistics are divided into two categories: drift, suspension, displacement, and settlement rate when the water flow is stable; and the critical parameters of state transition, such as critical suspension velocity, starting velocity, and resuspension velocity.

The experimental data were analyzed by Matlab 2023 and SPSS 28.0 for nonlinear regression analysis, and the relationship between critical flow rate and influencing factors was fitted to minimize residuals. Data collation and spatial analysis were completed with Origin 2022, Microsoft Excel, and ArcGIS 10.8. All experimental procedures were carried out in a closed environment, and plastic containers were avoided as much as possible. The utensils used were rinsed three times with ultrapure water and dried to reduce background pollution.

### 2.4. Overview of Study Area

The Mulanxi River, the largest river in central Fujian Province, China (118°38′~119°06′ E, 25°22′~25°25′ N), stretches 105 km with a drainage area of 1732 km^2^. Influenced by a subtropical monsoon climate, the river features rapid flow and abundant runoff, with an average annual discharge of 9.85 × 10^8^ m^3^. As a vital conduit to the sea and a key waterway for Putian City, the Mulanxi also serves as a major receptor of anthropogenic pollutants. Intensive human activities within its densely populated basin—particularly industrial and domestic wastewater discharges—have made the river a significant source of microplastic contamination, underscoring the need for systematic investigation of MP transport dynamics in this region.

**Figure 4:**
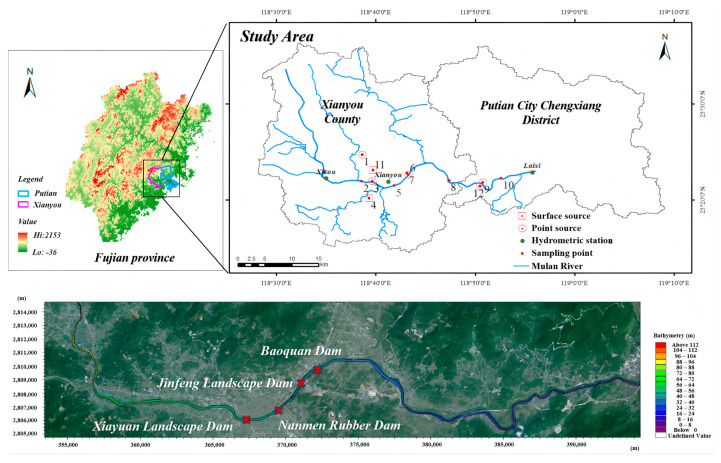
Model calculation area and sampling point layout diagram.

This study focuses on the Xianyou section of the Mulanxi River, spanning 46.55 km from Duwei Town (118.56° E, 25.44° N) to Laixi Hydrological Station (118.95° E, 25.38° N), and includes key river segments at Xitai, Shima, and Yuantou Bridges. Four dams—Xiayuan Landscape Dam (3.6 m), Nanmen Rubber Dam (4.1 m), Jinfeng Landscape Dam (3.9 m), and Baoquan Dam (3.0 m)—were incorporated as generalized hydraulic structures. The domain is divided into upstream (Duwei Town to Xiayuan Landscape Dam), midstream (Xiayuan Landscape Dam to Shima Bridge), and downstream (Shima Bridge to Laixi Hydrological Station). Based on measured terrain data, a gradient-based unstructured triangular mesh was constructed with 40,020 nodes and 72,753 grids over 54 km. To represent hydrological influences on MP transport, the model accounts for 8 tributary inflows and 7 point/non-point sources (Figure 4). Sampling points were classified into land, water, and sewage treatment types according to MP emission and occurrence patterns. A total of 13 representative points were sampled from 12 to 18 August 2022. The specific sampling point information is shown in Table 1. The GPS coordinates, the surrounding environment, and other information of each sampling point were recorded and photographed.

## 3. Model Input Condition Setting and Model Validation

### 3.1. Input Condition Setting

Model inputs included point sources configured from hydrological data (2010~2020) at Xianyou and Laixi hydrological stations, using explicit solutions of shallow water and transport equations. The typical year 2011 was selected based on Laixi flow records. Grid cells were classified as wet, semi-wet, or dry based on water depth thresholds for momentum and mass flux treatment, and bed roughness was represented by the Manning number (Table 2). Non-point sources were simulated using the Hydrological, Ecological, and Water Quality Model [36] (HEQM), and the measured data are input into HEQM and expressed as an equivalent point source in the coupled transmission framework.

Four MP types—PE, PS, PA, and PVC—were selected as simulation objects, divided into three size classes: small particle size (0.005~0.5 mm), medium particle size (0.5~1.7 mm), and large particle size (1.7~5 mm). MP shapes were distinguished by the Corey Shape Factor (*CSF*): particle/columnar (*CSF* ∈ (0.8, 1.2)) and fragment/film (*CSF* ∈ (0, 0.3)). Due to their complex and uncertain transport behavior, fibers were excluded from the simulations. Motion parameters from hydraulic experiments were averaged for each particle type to represent migration rates, accounting for rotation and turnover during transport.

### 3.2. Identify the Hydraulic Threshold for MP Movement Change

After entering the water body, MPs will show four different motion states of drift, suspension, moving, and rest due to the differences in density and other factors. When the external water flow conditions change to the critical velocity, the suspended mass in the water body will become a suspended state; the MPs resting at the bottom of the water body will begin to roll, slide, and undergo other movements; the bed load will appear in the suspension phenomenon. The migration coefficient (drift coefficient $k_{1}$ or suspension coefficient $k_{2}$) of different MPs under different motion states will be different. The measured critical flow rate also varies with the different types of MPs. Based on the results of hydraulic experiments, the migration coefficient ($k_{1}$ or $k_{2}$) and critical thresholds (critical suspension velocity, critical startup velocity $u_{s}$ and critical resuspension velocity $u_{r}$) Different MPs are identified and obtained as shown in Table 3.

### 3.3. Model Validation

**Figure 5:**
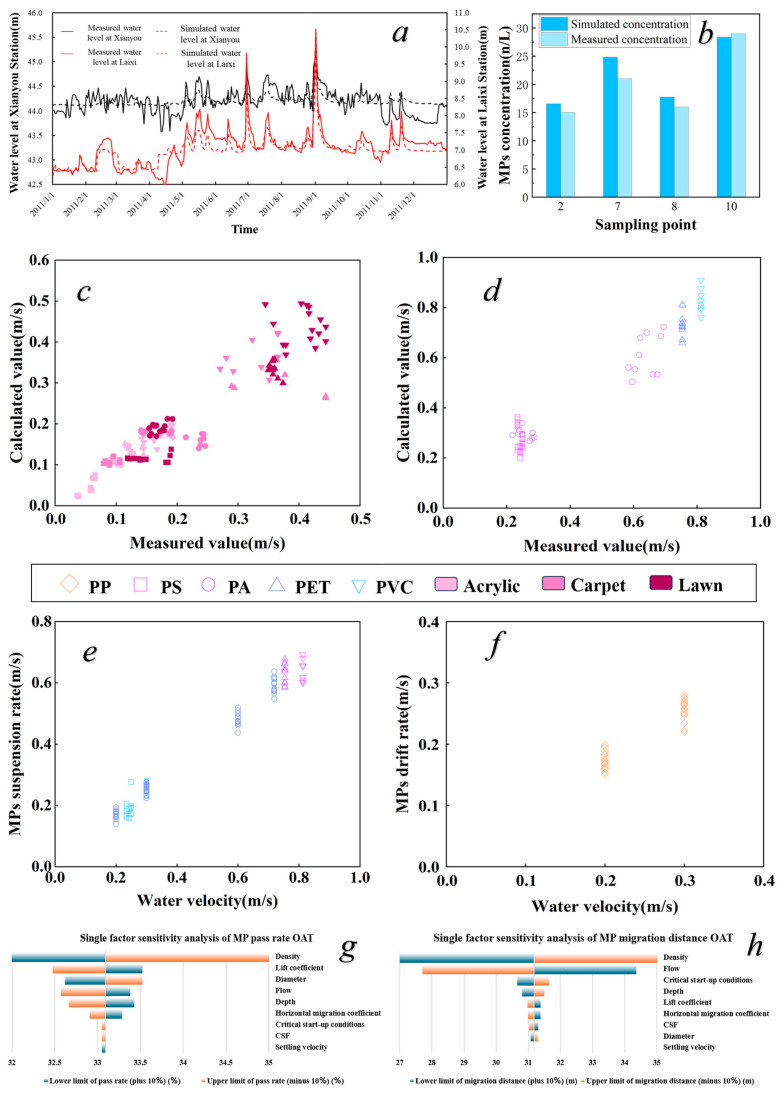
Model validation and hydraulic experiment part results ((**a**): hydrodynamic model validation diagram; (**b**): MP abundance simulation measured comparison diagram; (**c**): comparison of measured value and fitting of critical startup velocity (The filling color of the solid patterns represents the bottom condition of the corresponding MPs, e.g., the PS particle under acrylic bottom condition); (**d**): critical resuspension/suspension velocity measured value and fitting comparison; (**e**): resuspension/suspension coefficient after fitting; (**f**): fitted drift coefficient; (**g**): Single factor sensitivity analysis of MP pass rate OAT; (**h**): Single factor sensitivity analysis of MP migration distance OAT).

The hydrodynamic model was validated by comparing simulated and measured water levels at the Xianyou and Laixi hydrological stations, with the results shown in Figure 5a. The correlation coefficients (r) at these two stations were 0.71 and 0.90, with Nash–Sutcliffe efficiency (NS) values of 0.56 and 0.75, respectively. For MP abundance, simulated values were compared with field measurements at four sampling sites (Figure 5b), yielding r values of 0.83~0.86, Nbias of −0.04~−0.01, and NS of 0.60~0.67. The complete error statistics are summarized in Table 4. These metrics collectively confirm that the model accurately reproduces both hydrodynamic conditions and MP transport patterns, with no significant systematic bias.

According to the experimental results of the hydraulic experiment, the hydraulic formula is fitted, and the characteristic parameters are determined. In this study, the measured values of the critical startup velocity (Figure 5c), the critical resuspension velocity, and the critical suspension velocity (Figure 5d) were compared with the simulated values calculated by the formula. The fitting comparison showed that the slopes of the two fitting lines were 0.9964 and 0.98, respectively, indicating that the fitting effect was good. At the same time, the suspension coefficient (Figure 5e) and the drift coefficient (Figure 5f) are fitted experimentally, and the specific values are shown in Table 3.

The single factor sensitivity analysis (Figure 5g,h) identifies particle density as the most influential parameter controlling pass rate and migration distance, with higher density substantially increasing retention, while flow velocity emerges as the second key driver governing transport distance—highlighting the necessity of robust hydrodynamic validation. In addition to the density parameter, when the other parameters fluctuate by ±10%, the change range of the pass rate is not more than 1%, and the change range of the migration distance is not more than 4 m. This shows that the model has good stability in dealing with parameter uncertainty, and can still effectively reflect the influence of physical factors such as particle density and flow rate on system behavior.

## 4. Results and Discussion

### 4.1. Spatial Distribution and Characterization of MPs

**Figure 6:**
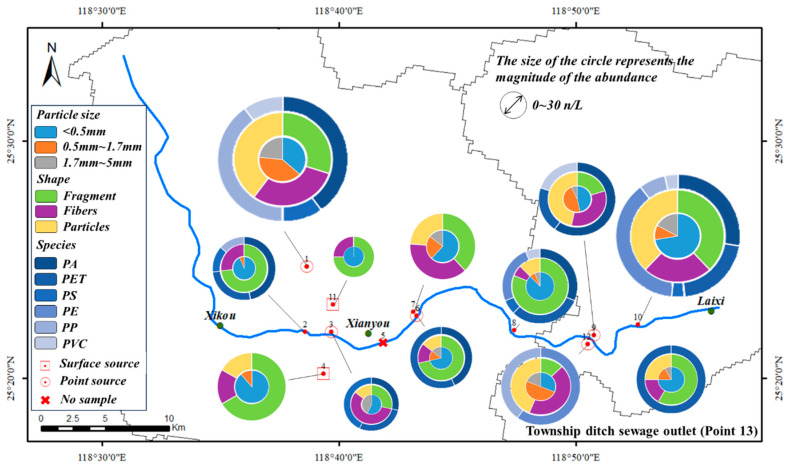
Measured distribution of MPs.

MPs were detected at all 13 sampling points (Figure 6), with abundances ranging from 7 to 30 n/L, showing significant spatial heterogeneity. The detailed findings are organized below by abundance and spatial distribution, particle size distribution, and shape and type composition.

#### 4.1.1. MP Abundance and Spatial Distribution

Abundance exhibited a clear increasing trend from upstream to downstream, with upstream agricultural and forested areas showing lower values (e.g., 7 n/L at sampling point 4) while downstream urban sections showed substantially higher values, reaching up to 30 n/L at the drainage outlet of the power plant (point 1) and Yuantou Bridge (point 10). The lowest abundances (7 n/L) were recorded at the machinery factory sewage outlet (point 3) and Sewage treatment plant outfall (point 6). Overall, MP abundance in river water ranged from 14 to 24 n/L, consistent with the cumulative effect of point sources along the urbanized middle and lower reaches, where sewage treatment plants directly input large MP loads [37]. This downstream enrichment aligns with observations in other coastal rivers in China, highlighting rivers as key transport channels for MPs from land to sea [38].

#### 4.1.2. Particle Size Distribution

Small-sized MPs (<0.5 mm) dominated both pollution sources (64.04%, 57 n/L) and water samples (71.62%), with abundance negatively correlated with particle size. Notably, sampling points 11 (Refuse landfill) and 4 (Agricultural greenhouse) showed nearly complete dominance of small particles, reaching 100% and 88.9%, respectively. The predominance of small particles reflects continuous fragmentation of larger plastics through physical, chemical, and biological degradation during transport [39]. The Mulanxi River’s fast-flowing, short-residence hydrological regime, typical of mountain-plain transitional rivers, likely accelerates mechanical wear of plastics, consistent with findings from Danxia landform urban rivers where hydrodynamics and topography jointly shape the MP size spectrum [40].

#### 4.1.3. Shape and Type of MPs

Regarding MP shape and type, pollution sources exhibited a relatively balanced composition—particles (34.8%), fragments (38.2%), and fibers (27.0%)—whereas fibers dominated water samples at 37.8%, reflecting their preferential transport over long distances owing to small size and light weight [41], while particles and fragments are more readily retained by dams and river bends. This hydrodynamic sorting is likely amplified by the dense dam network in the middle and lower reaches, promoting fiber enrichment in the water column [42]. Type analysis identified PE, PA, PVC, and PP as the main components, with PE being the most abundant (30.51%), consistent with their widespread daily use [43]. Notably, no specific MP types were detected at sites 4, 7, and 11, possibly due to mixing or environmental degradation. The spatial distribution of MP types reflects a combination of domestic, industrial, and agricultural inputs—PE and PP mainly from packaging and agricultural films, and PVC from construction materials [44]—a pattern comparable to other urban rivers in China, such as the Pearl River Basin, highlighting the dominant role of urban consumption in shaping MP type profiles [45].

### 4.2. Analysis of Motion Characteristics of MPs

Based on the model simulation results, the migration trajectories of MPs after release from different point sources and non-point sources were plotted.

**Figure 7:**
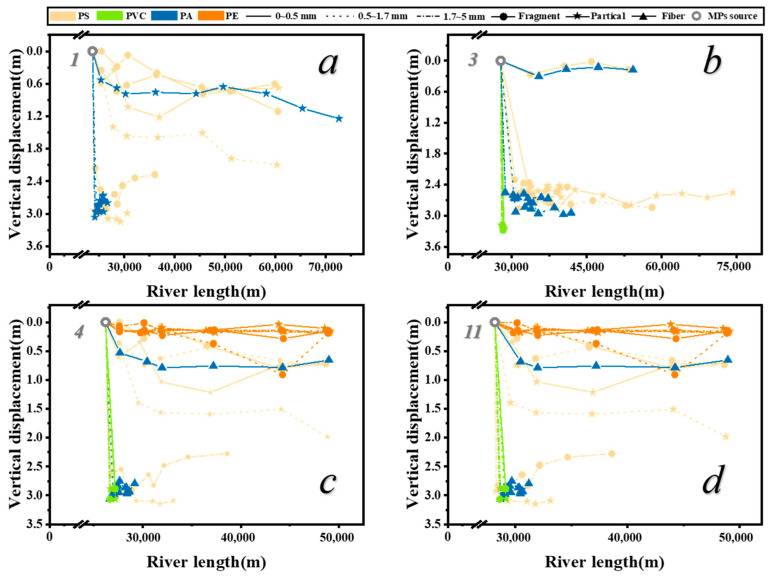
Migration trajectory of MPs at different sampling points. ((**a**): point source sampling point 1; (**b**): point source sampling point 3; (**c**): non-point source sampling point 4; (**d**): non-point source sampling point 11).

Based on the model simulation results, the migration trajectories of MPs released from typical point sources (sampling points 1 and 3) and non-point sources (sampling points 4 and 11) were plotted (Figure 7). In the simulation of non-point source pollution, because no specific MP type was detected, the whole type of MPs was simulated. The results show that the MPs released from point source and non-point source pollution are similar in migration trajectory, but their final destination is regulated by their own physical properties and hydrodynamic conditions.

In the vertical direction, the density of MPs is a key factor in determining their stratification in water. PE with a density less than water mainly migrates in the upper water body in the form of drift in all shapes and particle sizes. The density of PS is slightly larger than that of water, and the small particle size part can be suspended and migrated, while the large particle size part is easy to settle in the middle and lower layers of the water body in a suspended or moving state. The behavior of PA is similar to that of PS, but PA with a particle size greater than 0.5 mm is more inclined to moving at the bottom of the riverbed. It is worth noting that the movement of the PA fiber shows high uncertainty, which is related to the change in center of gravity and uneven force caused by its special shape. High-density PVC will rapidly settle in still water or at low flow rates; only when the hydrodynamic conditions are enhanced to a certain extent, PVC particles may jump or even resuspend [27]. This series of density-based stratification rules confirms that hydrodynamic force is the core driving force to control the distribution of MPs with different densities between the riverbed and water column [21].

In the horizontal direction, the lateral migration distance of MPs with small particle size and low density is farther, and the bottom rolling and resuspension are more likely to occur [46]. The shape of MPs significantly affects the migration rate: in the suspended state, the migration rate of fibers is the fastest, followed by fragments and particles; when the bottom is pushed, the migration rate of particle MPs is the fastest. The simulation shows that PE as drift mass and small particle size PS and PA as suspended mass can move out of the study area with water flow, while high-density PVC is difficult to output due to easy sedimentation [47]. This difference in movement revealed by this study highlights the need to identify complex migration pathways of MPs at the watershed scale [48].

The random motion of MPs (such as rotation and rolling) observed in the hydrodynamic experiment is consistent with the simulation results. The motion state of MPs is the result of the interaction of density, shape, and particle size: for drift mass and bed load, density plays a leading role; for suspended sediment, its state is determined by both density and shape [49]. It is worth noting that the fiber exhibits a faster migration rate and higher random motion frequency due to its special shape, which leads to better reliability of the model for the simulation of fragments, particles, and films, but the characterization of fiber motion still faces significant challenges. Future research needs to further couple environmental processes such as ultraviolet aging and biofilm attachment. For example, ultraviolet aging can change the surface properties and density of MPs, affecting their aggregation and sedimentation [50]; clay minerals or natural organic matter (such as humic acid) in water can accelerate their migration and transformation by promoting aggregation or biological sedimentation [51]. Therefore, to fully understand the migration mechanism of MPs in natural rivers such as the Mulanxi River, it is necessary to comprehensively consider the synergistic effects of their physical properties, hydrodynamic conditions, and complex environmental geochemical processes.

### 4.3. Prediction of MP Enrichment Characteristics

**Figure 8:**
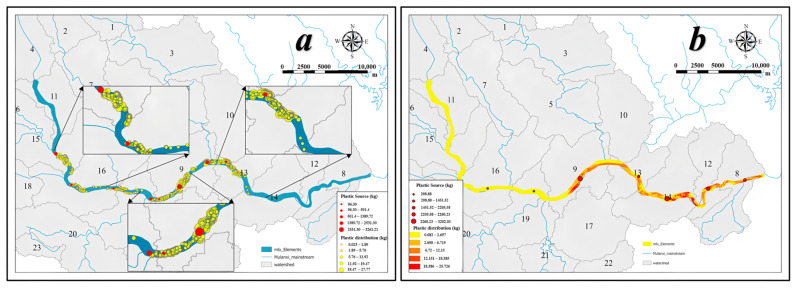
MP enrichment characteristics ((**a**): point source; (**b**): non-point source).

The MP mass distribution map (Figure 8) based on the model simulation revealed that the enrichment of MPs in the Mulanxi River Basin showed significant spatial heterogeneity, which was comprehensively regulated by the type of pollution source, hydrodynamic conditions, and topography.

Non-point source pollution has a fundamental impact on the distribution of MPs in the whole river section, especially in the downstream, with a wide range of input fluxes (298.88–3208.08 kg). This is mainly due to the input of broken plastic waste, tire wear particles, etc., through surface runoff and atmospheric deposition [52,53]. MPs from these sources generally have the characteristics of small particle size and low density, and are easy to migrate. Their spatial distribution is closely related to the intensity of human activities in the basin [54]. For example, the upper reaches of the Mulanxi River (such as sampling points 4 and 11) have gentle terrain, weak human activities, and low MP deposition (only 597.76 kg). In the middle and lower reaches of the urban area, dense urban surface runoff has become an important non-point source input. Recent studies have confirmed that urban rainfall runoff is a key channel for MPs to enter the water body, and its emission characteristics are significantly affected by land use types and rainfall intensity [55,56]. In addition, short-term fertilization in agricultural activity areas (near sampling point 4) may also change the emission fluxes and characteristics of MPs in surface runoff [57].

Point source pollution dominates the local high-intensity enrichment of the downstream river section, and the input is between 96.30 and 3261.21 kg. The downstream accumulation of MPs released from point sources such as sewage treatment plants, industrial and domestic sewage outlets is positively correlated with their emission intensity, confirming the direct impact of human activities on the output of MPs [58]. MPs with high density and large particle size are more likely to settle at a close distance downstream of the discharge outlet, while low-density particles may continue to migrate with the water flow. Water conservancy facilities such as Chengguan Dam and Jinfeng Bridge Dam in the middle reaches of Mulanxi River promote the enrichment of particles and fragments by intercepting water flow and reducing flow velocity, which is consistent with the MP interception effect observed in other dam-type reservoirs [59]. In addition, field observations found that at the significant river bend between sampling points 7–8, the centrifugal force of water flow led to the enrichment of MPs on the convex bank outside the river, which further reflected the secondary regulation of natural topography on the spatial distribution of MPs.

In summary, the enrichment pattern of MPs in the Mulanxi River is the result of the interaction of the source-sink process and migration path. Non-point sources provide wide-area and continuous non-point source input, and their distribution is controlled by the urbanization of the basin and the macro-control of land use patterns [60]. The point source forms a high-intensity ‘hot spot’ locally, and its influence range is constrained by the emission characteristics and downstream hydrodynamic conditions. The hydraulic structures (such as dams) and special terrain (such as river bends) in the river channel become the key nodes to control the final destination of MPs by changing the local hydrodynamic force. This ‘source-path-sink’ coupling mechanism makes it easier for high-density, large-size MPs to be buried long-term in the middle reaches of the dam and the bottom of the gentle urban river [61], while lightweight, small-size MPs have higher potential for downstream and estuary output. Therefore, the effective control of river MP pollution needs to consider the comprehensive strategies of source emission reduction, process interception, and end treatment according to the dominant driving mechanism of different river sections.

### 4.4. Prediction Analysis of MP Transport Flux

In this study, typical pollution sources were selected. Based on the sample data of the dry season (January–May, December) and wet season (June–November) in 2022, combined with the river inflow coefficient of 0.8, the river inflow and transport flux of MPs in the Mulanxi River were quantified. Since no specific MPs were detected in Agricultural greenhouses (source 4) and Refuse landfill (source 11), this study did not predict the flux of non-point sources.

The simulation results (Table 5 and Table 6) show that the transport flux of MPs in the Mulanxi River has significant spatial differences and hydrological driving characteristics. The annual input of each point source is 1.84 × 10^8^–6.71 × 10^11^ n, and the annual output is 1.70 × 10^8^–4.39 × 10^11^ n. The total point source transport rates in the dry season and the wet season were 32.20% and 36.14%, respectively, indicating that the regulation of hydrological seasonality on the overall output efficiency was relatively limited. This limited seasonal difference may be the result of competition between ‘hydrological dilution effect’ and ‘biological sedimentation effect’ [62,63]: Although the increase in runoff in the wet season enhances the hydrodynamic transport potential, it also dilutes the concentration of MPs, and may promote the formation of biofilms due to the increase in nutrient concentration, accelerating the deposition of MPs [64], which partially offsets the transport contribution of increased flow.

The physical properties of MPs are the inherent key to determining their migration ability and output efficiency. This study found that small particle size and fibrous MPs were more likely to migrate out of the study area. Pollution source 3 (Machinery factory sewage outlet) maintained a high transport rate (45.81% in dry season and 46.61% in wet season) despite the lowest input and remote location, which was attributed to the fact that the released MPs were dominated by low-density and small-sized fibers/fragments. On the contrary, the transport rate of large-sized MPs in pollution source 6 and pollution source 9 (both of them are Sewage treatment plant outfall) is zero in the dry season and is completely intercepted, which is related to the lack of hydrodynamic force, which leads to insufficient drag force and lift force to maintain its suspension [65]. This indicates that the physical characteristics (density, shape, and particle size) of MPs emitted by pollution sources have a decisive influence on their fate in the watershed. This conclusion is consistent with other studies indicating that the type and shape of MPs are key parameters for predicting MP transport fluxes [66].

From the perspective of watershed management, the total output flux of MPs (annual output of 9.37 × 10^11^ n/a, total transport rate of 68.34%) in the Mulanxi River shows that it has significant potential for downstream and marine output. This flux level is comparable to estimates of other rivers around the world [67], emphasizing the dual role of urban rivers as ‘source’ and ‘transport channel’ of MPs. It is worth noting that the transport rate predicted by the model did not increase significantly due to the sharp increase in runoff in the wet season. This means that it is difficult to effectively reduce the long-term output load of MPs only by controlling terminal emissions or relying on seasonal hydrological erosion. Future pollution control strategies should pay more attention to source emission reduction, especially for MPs with high mobility (low density, small particle size, fibrous). For example, improving the removal efficiency of fibrous MPs in sewage treatment plants [68], as well as comprehensive treatment from non-point sources such as urban surface runoff [69] and agricultural non-point sources [70,71], is essential to block the input of MPs into freshwater ecosystems.

## 5. Limitations

While this study provides a mechanistic framework for simulating multi-type MP transport in riverine systems, several limitations should be acknowledged. Firstly, fibrous MPs were excluded from the numerical simulations due to their complex and highly stochastic motion arising from shape-induced variations in center of gravity and asymmetric force distribution, which complicates accurate model parameterization; future model development incorporating shape-specific drag coefficients and tumbling dynamics would be necessary to better capture fiber transport. Secondly, the two-dimensional depth-averaged hydrodynamic framework cannot resolve vertical variations in flow velocity, turbulence, and MP concentration, which is particularly relevant for density-dependent stratification behaviors, while a three-dimensional model with vertical turbulence closure would provide more accurate predictions. Thirdly, the hydraulic parameters—including critical velocities and migration coefficients—were derived from idealized flume experiments that may not fully represent the complex bed roughness, sediment heterogeneity, and turbulent structures in natural rivers, and field-scale calibration with more extensive datasets would further enhance their generalizability. Despite these limitations, the model demonstrates robust predictive performance for fragments, particles, and films, as confirmed by field validation, and its mechanistic foundation renders it well-suited for future refinements.

## 6. Conclusions

In this study, a multi-type MP migration model driven by river hydrodynamic conditions was developed by integrating hydraulic experiments, field investigations, and particle tracking simulations. The main findings are summarized as follows:

(1) The model incorporates individual differences in MP properties (density, shape, and size) into force-based motion analysis, enabling accurate simulation of migration pathways for diverse MP types. Validation against hydraulic experiments and field measurements confirmed that MP movement is governed by the combined effects of particle density, shape, and size.

(2) Field sampling revealed that MP abundance in the Mulanxi River ranged from 14 to 24 n/L, with particles smaller than 0.5 mm accounting for the highest proportion (71.62%). Fragmentary and fibrous morphologies dominated the MP community.

(3) Model simulations demonstrated that total transport rates of typical pollution sources ranged from 20.7% to 47.33% in the dry season and 21.26% to 46.82% in the wet season. The total annual flux reached 9.37 × 10^11^ particles, with an overall transport rate of 68.34%, indicating strong export potential of MPs from the Mulanxi River Basin. Seasonal hydrological changes exerted a relatively limited influence on overall output efficiency.

(4) The model exhibits strong versatility and adaptability to various freshwater systems, providing a mechanistic foundation for predicting MP transport in riverine environments. Future refinements incorporating three-dimensional hydrodynamic frameworks and environmentally relevant aging processes would further enhance predictive accuracy.

## Figures and Tables

**Table 1:** Sampling point information table.

No.	Sampling Coordinate	Sampling Point Position	Remark
1	118.644° E; 25.412° N	Drainage outlet of the power plant	Waste treatment plant (Point source)
2	118.643° E; 25.366° N	Xitai Bridge	Water quality testing site (Water body)
3	118.661° E; 25.366° N	Machinery factory sewage outlet	Industrial sewage outlet (Point source)
4	118.656° E; 25.337° N	Agricultural greenhouse	Agricultural emission source (N-Point source)
5	118.709° E; 25.361° N	Chengguan Dam	Sluice front (Water body)
6	118.721° E; 25.378° N	Sewage treatment plant outfall	Sewage treatment plant (Point source)
7	118.730° E; 25.383° N	Jinfengqiao Dam	Sluice front (Water body)
8	118.790° E; 25.367° N	Shima Bridge	Water quality testing site (Water body)
9	118.846° E; 25.364° N	Sewage treatment plant outfall	Waste treatment plant (Point source)
10	118.877° E; 25.371° N	Yuantou bridge	Water quality testing site (Water body)
11	118.662° E; 25.386° N	Refuse landfill	Waste treatment plant (N-Point source)
12	118.842° E; 25.358° N	Village outfalls (pipes)	Township sewage outlet (Point source)
13	118.842° E; 25.358° N	Village outfalls (ditches)	Township sewage outlet (Point source)

**Table 2:** Main parameters of the MIKE hydrodynamic model.

Model Parameter	Values	Dimension
CFL	1	/
Time step	1	h
Dry water depth	0.005	m
Semi-humid water depth	0.05	m
Wet water depth	0.1	m
Manning number	0.28	m^1/3^/s
Coefficient of eddy viscosity	0.0000018~10^10^	m^2^/s
Coriolis Forcing	0	/
Wind Forcing	0	/
Ice Coverage	0	/
Precipitation-Evaporation	0	/

**Table 3:** Migration coefficient and critical velocity value (measured).

Type	Horizontal Migration Coefficient ($k_{1}$ or $k_{2}$)		Critical Suspension Velocity (m/s)		Critical Startup Velocity (m/s)	
	Nonfibrous	Fibroid	Nonfibrous	Fibroid	Particles	Fragments
PE	0.91					
PS	0.86		0.251		0.059	0.065
PA	0.84	0.94	0.649	0.430	0.090	0.105
PET	0.84		0.705		0.143	0.188
PVC	0.85		0.710		0.138	0.190

Note: Experimental observations indicate that the critical suspension velocity equals the critical resuspension velocity and is independent of bed conditions; hence, the two are treated as identical in formula fitting. For non-fibrous MPs (particles and fragments), the horizontal migration coefficient and critical suspension rate are fitted using a unified method due to similar data characteristics, whereas fibrous MPs exhibit notably different behavior.

**Table 4:** Error metrics for model validation at hydrological stations and MP sampling points.

Sampling Point	Nbias	r	NS
Xianyou Hydrological Station	0.00	0.71	0.56
Laixi Hydrological Station	0.01	0.90	0.75
Xitai Bridge	−0.04	0.85	0.62
Jinfengqiao Dam	−0.04	0.83	0.6
Shima Bridge	−0.04	0.85	0.67
Yuantou bridge	−0.01	0.86	0.66

**Table 5:** The input and migration quantity of MP from pollution sources in the Mulanxi River.

Pollution Source	Quantity into the River (n/a)	Fluxes in Different Seasons (n)		Total Flux (n/a)
		Dry Season	Wet Season	
1	6.71 × 10^11^	1.92 × 10^11^	2.46 × 10^11^	4.39 × 10^11^
3	1.84 × 10^8^	8.43 × 10^7^	8.58 × 10^7^	1.70 × 10^8^
6	1.66 × 10^11^	3.42 × 10^10^	3.53 × 10^10^	6.95 × 10^10^
9	1.66 × 10^11^	7.86 × 10^10^	7.77 × 10^10^	1.56 × 10^11^
12/13	3.68 × 10^11^	1.36 × 10^11^	1.36 × 10^11^	2.72 × 10^11^
Total flux (n)	1.37 × 10^12^	4.41 × 10^11^	4.96 × 10^11^	9.37 × 10^11^

**Table 6:** Estimation of MP transport rate of pollution sources in different seasons in Mulanxi River.

Different Seasons	Pollution Source	Percent of Pass (%)					Total Transport Rate (%)	
		Particle Size (mm)			Shape			
		0~0.5	0.5~1.7	1.7~5	Particle	Fiber/ Fragment	Pollution Source	Total Season
Dry season	1	48.88	30.44	11.05	9.02	37.12	32.68	32.20
	3	70.98	53.47	12.00	34.04	50.37	45.81	
	6	27.67	6.53	0.00	11.02	44.58	20.70	
	9	54.60	0.00	0.00	2.71	58.48	37.00	
	12/13	42.97	30.32	30.00	36.38	37.34	47.33	
Wet season	1	49.68	38.00	14.14	9.79	42.14	36.72	36.14
	3	71.27	53.88	13.49	34.87	51.30	46.61	
	6	28.41	6.65	0.12	11.43	45.51	21.26	
	9	54.60	45.12	4.32	2.71	57.85	37.00	
	12/13	42.97	30.32	30.00	37.34	37.34	46.82	

## Data Availability

The original contributions presented in this study are included in the article. Further inquiries can be directed to the corresponding author.
